# Genetic and Morphological Analyses of Native Vanilla Populations in Mexico Call into Question the Taxonomy of *V. odorata*

**DOI:** 10.3390/plants15111661

**Published:** 2026-05-28

**Authors:** Eduardo Peña-Mojica, Rinah H. Ravelonanosy, José B. Azofeifa-Bolaños, Frank Solano-Campos, Carine Charron, Félicien Favre, Michel Grisoni, Madeleine Hidalgo-Morales, Araceli Pérez-Silva

**Affiliations:** 1 Unidad de Investigación y Desarrollo en Alimentos (UNIDA), Tecnológico Nacional de México-Veracruz, Veracruz 91860, Veracruz, Mexico; epmojica09@hotmail.com; 2 Departamento de Ingeniería Química y Bioquímica, Tecnológico Nacional de México-Tuxtepec, San Juan Bautista Tuxtepec 68350, Oaxaca, Mexico; 3 University of la Réunion, UMR PVBMT, 97410 Saint-Pierre, La Réunion, France; harinomena-rinah.andrianarivelo@univ-reunion.fr; 4 Laboratorio de Biotecnología de Plantas, Escuela de Ciencias Biológicas, Universidad Nacional, Heredia 40101, Heredia, Costa Rica; bernal.azofeifa.bolanos@una.cr (J.B.A.-B.); frank.solano.campos@una.ac.cr (F.S.-C.); 5 Herbario Anastasio Alfaro González (AAG), Escuela de Ciencias Biológicas, Universidad Nacional, Heredia 40101, Heredia, Costa Rica; 6 CIRAD, UMR PVBMT, St Pierre F-97410, Réunion; carine.charron@cirad.fr; 7 CIRAD, UMR PVBMT, Toamasina 501, Madagascar; felicien.favre@cirad.fr (F.F.); michel.grisoni@laposte.net (M.G.)

**Keywords:** vanilla, *Vanilla odorata*, integrative taxonomy, morphology, phylogenetic analysis, Genotyping by Sequencing

## Abstract

Wild vanilla populations with high aromatic potential and morphological affinity to *Vanilla odorata* have been identified in Oaxaca, Mexico. This study employed morphologic and molecular taxonomic approaches to characterize plant material collected from the field and subsequently maintained under uniform conditions at the Tecnológico Nacional de México Tuxtepec campus germplasm bank. Morphological characterization of the three populations was conducted, and genetic analyses of 155 accessions (2365 SNPs) were performed using Genotyping by Sequencing. The morphological analyses revealed clear differences in vegetative and reproductive traits among the studied accessions. Phylogenetic and STRUCTURE analyses identified three groups within the *V. odorata*-like clade: a pure group, and two hybrid groups involving *V. odorata* and *V. insignis* or *V. cribbiana*. These findings reveal a high level of phenotypic and genetic diversity within wild vanilla populations from Oaxaca and suggest that hybridization may have played an important role in the evolutionary history of the group. Furthermore, the results challenge the current taxonomic circumscription of *Vanilla odorata*, indicating that its taxonomic identity and evolutionary origin require re-evaluation. This study provides new insights into the diversification and taxonomy of aromatic wild vanilla species in Mesoamerica.

## 1. Introduction

*Vanilla* is an ancient genus within the family Orchidaceae that encompasses a highly diverse group of species that are widely distributed throughout the tropics between 27° north and south, except in Australia [1,2]. Soto-Arenas and Cribb [3] classified this group into two subgenera, *Vanilla* (membranous leaves) and *Xanata* (non-membranous leaves), and further divided *Xanata* into two sections: *Xanata* (American species) and *Tethya* (West Indian and Old-World species). To date, approximately 140 *Vanilla* species have been proposed, most of which originated in the American continent [4,5]. However, it has been suggested, based on alpha-taxonomy, that several recently described species represent synonyms of previously named taxa, reducing the number of accepted species [5]. Therefore, a thorough revision of the taxonomic classification of *Vanilla* species is needed. However, such a task is challenging as the genus is considered a complex taxonomic group that exhibits uniparental reproduction, interspecific hybridization in sympatric areas, and polyploidy [1,4]. Consequently, species identification within *Vanilla* requires the integration of both classical and modern taxonomic approaches as well as ecological, reproductive biology, cytogenetic, and genetic analyses [2,6,7,8]. Recently, a revision of the nomenclature and some modification of the subgeneric classification of the genus *Vanilla* was proposed [9]. The authors identified 130 species distributed among four subgenera and seven sections. A key implication of this updated framework is the reclassification of the aromatic species group, which is now nested within subgenus *Vanilla*, section *Vanilla*.

Morphological analysis plays a key role in the identification and discrimination of species, including within the genus *Vanilla*. Numerous studies have conducted morphometric analyses of different plant structures, including leaves, stems, flowers, and fruits [10,11,12,13,14,15]. However, they are often insufficient to unambiguously delineate closely related species. Nonetheless, the guidelines published by the International Union for the Protection of New Varieties of Plants (UPOV) [16] provide a set of qualitative and quantitative descriptors for fruits, leaves, stems, and flowers to differentiate varieties of commercial vanilla species and hybrids.

While traditional alpha-taxonomy provides the morphological framework for the recognition and description of vanilla species, molecular phylogenetic studies have substantially transformed the way vanilla and its related taxa are currently understood. The phylogenetic position of vanilla within the family Orchidaceae has become clearer due to numerous plastid DNA studies [1,17,18,19,20]. The use of molecular markers such as ITS or plastid sequences, which have been applied for the genetic analysis of species in the *Vanilla* genus in Mexico, has only allowed for the separation of species groups [21,22] but not closely related species such as *Vanilla odorata* C. Presl, *Vanilla karen-christianae* Karremans & P. Lehm, *Vanilla helleri* A.D. Hawkes, and *Vanilla insignis* Ames. Currently, genomics-based techniques, such as Genotyping by Sequencing (GBS), have become powerful, high-throughput and cost-effective methods for determining relationships beyond a species-level resolution [6,7,8,23,24].

*Vanilla odorata* C. Presl is a species with a broad distribution across tropical America, and has been recorded in Mexico, Guatemala, Belize, Honduras, Nicaragua, Costa Rica, Panama, Colombia, Ecuador, Peru, Bolivia, and Brazil. This species was described by Karol Presl in 1826 [25] from incomplete specimens collected years earlier by Thaddeus Haenke in Guayaquil, Ecuador. However, the taxonomic delimitation of *V. odorata* remains challenging as several lectotypes were proposed for the species [5], and its foliar morphology (particularly the long, narrow shape of its leaves) is shared with several closely related species, including *Vanilla insignis* Ames, *Vanilla helleri* A.D. Hawkes, *Vanilla karen-christianae* Karremans & P. Lehm, *Vanilla ensifolia* Rolfe, *Vanilla uncinata* Huber ex Hoehne, *Vanilla fimbriata* Rolfe, *Vanilla denticulata* Pabst, and *Vanilla labellopapillata* A.K. Koch, Fraga, J.U. Santos & A.L. Ilkiu-Borg [3,5,25,26,27,28]. This morphological convergence has led to ambiguous identifications and a long history of controversial synonyms.

In Mexico, three wild populations morphologically related to *Vanilla odorata* and characterized by highly aromatic fruits have been identified in the Chinantla region of Oaxaca [26]. However, these populations also exhibit morphological variation and differential responses to environmental stress conditions, raising questions regarding their taxonomic identity and evolutionary relationships. Although these populations have traditionally been associated with *V. odorata* based primarily on foliar morphology, it remains unclear whether they correspond to *V. odorata sensu stricto*, represent distinct evolutionary lineages, or originated through hybridization with related species. Consequently, the present study employed a dual taxonomic approach coupling morphological and phylogenetic methods to (i) evaluate the morphological and genetic differences between the studied populations, (ii) clarify their phylogenetic relationships with closely related *Vanilla* species, and (iii) evaluate the evidence for genetic admixture in the studied populations and related species. Given the commercial potential of *V. odorata* and its inclusion in the Codex Alimentarius standards for vanilla [29], the utilization of its genetic resources represents a strategic opportunity to diversify the natural flavoring market, which is currently dominated primarily by *Vanilla planifolia* Jacks. ex Andrew and *Vanilla x tahitensis* J.W. Moore [30,31,32].

## 2. Results

### 2.1. Morphological Differentiation

**Figure 1:**
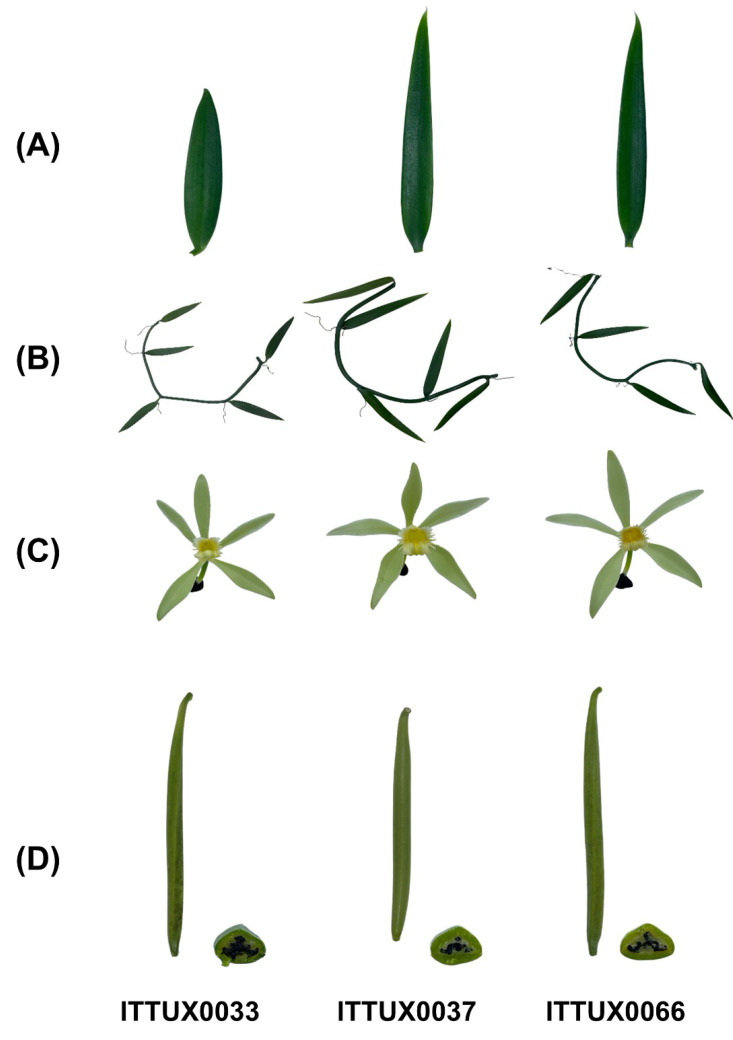
Representative images of the three Oaxaca odorata-like accessions. (**A**) Leaves, (**B**) stems, (**C**) flowers, and (**D**) fruits.

The morphological characteristics of the leaves, stems, flowers, and fruits of the three Oaxaca odorata-like accessions (ITTUX0033 (Santiago Tlatepusco), ITTUX0037 (Rancho Gavilán), and ITTUX0066 (San Isidro Naranjal)) are summarized in Table 1 and Figure 1. In addition, morphological datasheets were created for each accession (Figures S1–S3).

In all the accessions, the leaves presented a lanceolate shape and had a tapering base. The shape was similar to that of the UNA-VAN-00045 and UNA-VAN-00046 *V. odorata* accessions from Costa Rica. The apex shape was different in the ITTUX0033 accession (obtuse) compared to the ITTUX0037 and ITTUX0066 accessions (acute). The mean leaf length ranged from 14.94 to 19.96 cm, while the mean leaf width ranged from 2.16 to 2.43 cm. The leaf length differed significantly between the accessions, while statistically significant differences in leaf width were only observed between ITTUX0066 and ITTUX0033. ITTUX0037 had the longest leaves, while ITTUX0033 had the shortest and widest leaves (Figure 1A). The L/W ratio in ITTUX0066 was significantly higher compared to ITTUX0033 but not ITTUX0037. The lowest L/W ratio was obtained for ITTUX0033, confirming that it has the broadest leaves.

The stems of the three accessions are terete with a smooth surface. All accessions showed significant differences in internode length, while the stem diameter of ITTUX0037 was the thickest and significantly different from that of the two other accessions (Figure 1B). The diameter and internode distance of the stems allowed for the separation of accessions ITTUX0066 and ITTUX0037.

A total of 18 descriptors for flower morphology were analyzed, and significant differences in ten descriptors were observed in the Oaxaca odorata-like accessions. The samples exhibited differences in their main floral structures, such as the ovary, sepals, petals, labellum, and column. Accession ITTUX0033 exhibited the lowest values for FW, OWE, OL, LW, ASW, RLSW, PW, and RL, which were statistically significantly different from those of the other two accessions. Accession ITTUX0066 showed the highest values for these descriptors. However, the highest values for the labellum and column (LL and CL) were observed in accession ITTUX0033. The anther and rostellum (RW, AL, and AW) of the studied accessions did not show significant differences. In addition to the morphometric differences in the flowers of the *V. odorata*-related accessions, contrasting line colors were observed on the inner surface of the lip, ranging from yellow in ITTUX0033 to orange/brown in ITTUX0066, with an intermediate tone in ITTUX0037. The morphological differences among the flowers of the Oaxaca odorata-like accessions are illustrated in Figure 1C.

The morphometric values of the Oaxaca odorata-like accessions in this study and those reported in the literature for species related to *V. odorata* are presented in Table 2. The morphological comparisons revealed relationships between accession ITTUX0033 and *V. fimbriata*, as well as between ITTUX0037 and *V. ensifolia*.

In the three accessions, the ripe fruits exhibited an oblong shape and a triangular cross-section, as shown in Figure 1D. The fruits ranged from 15.66 to 19.68 cm in length and from 11.30 to 12.11 mm in width, and the weight ranged from 8.20 to 10.97 g. The largest fruits were obtained from accession ITTUX0066, while the smallest fruits were from accession ITTUX0037.

### 2.2. Genetic Differentiation

**Figure 2:**
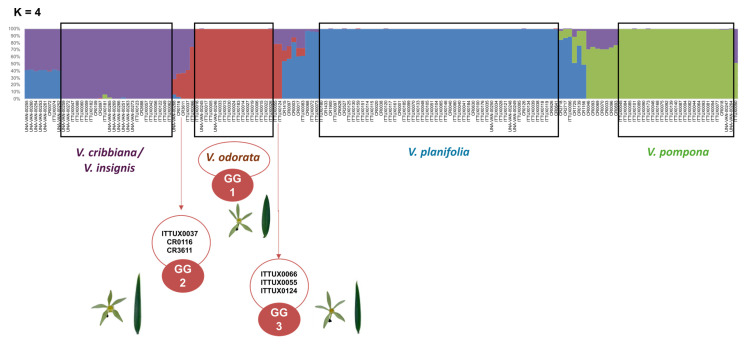
Genetic clustering analysis (STRUCTURE) (K = 4) of 155 accessions from different collections: ITTUX (Mexico), UNA (Costa Rica), and BRC Vatel (Reunion Island). The red circles indicate the different genetic groups of the Oaxaca odorata-like accessions. Colors represent the following taxa: *V. cribbiana/V. insignis* (purple), *V. odorata* (red), *V. planifolia* (blue) and *V. pompona* (green).

The STRUCTURE analysis of 155 accessions from different collections—from Mexico, Costa Rica, and Réunion Island—was performed using 2365 SNPs (Figure 2). According to the Evanno method, the highest estimated probability was for K = 4 [33] (Figure S4). This result suggested the presence of four groups corresponding to the following species: *V. cribbiana*/*V. insignis*, *V. odorata*, *V. planifolia*, and *V. pompona*. The high genetic diversity within the studied accessions facilitated the demarcation of populations related to *V. odorata*.

**Figure 3:**
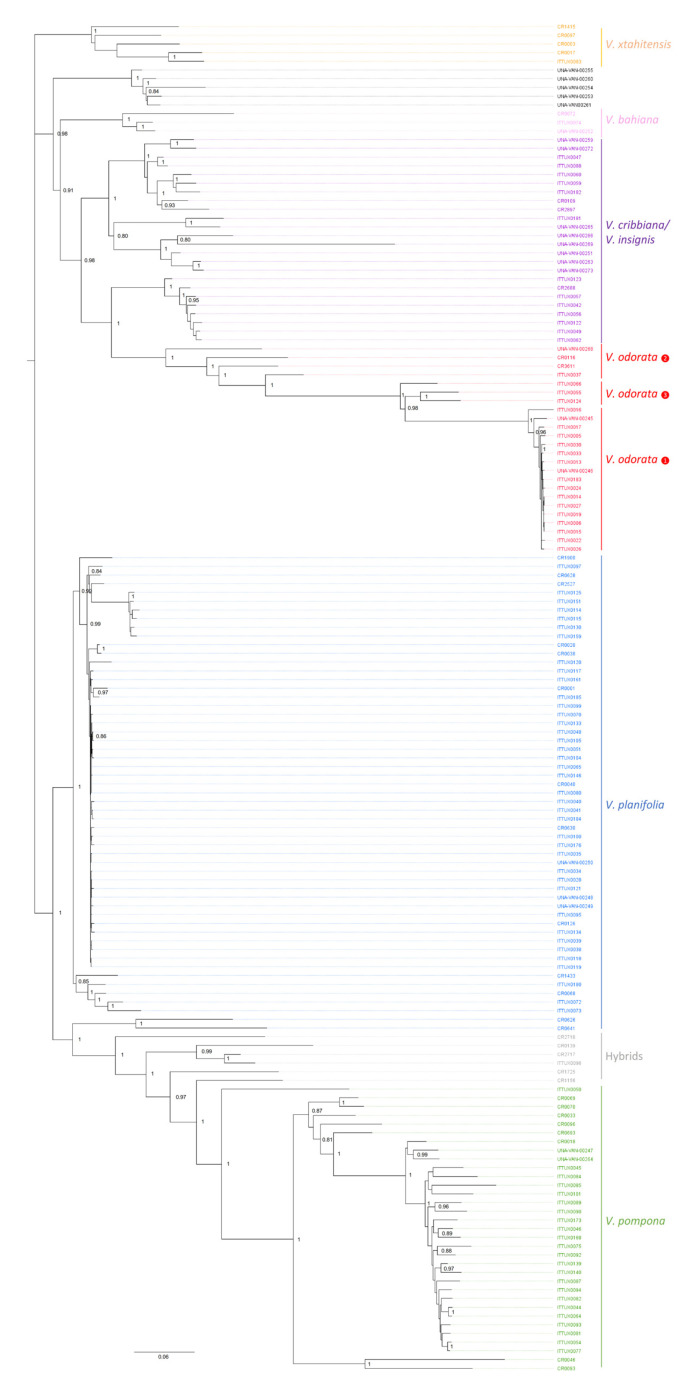
Phylogenetic relationships among vanilla accessions inferred using an unweighted Neighbor-Joining (NJ) tree based on 2365 SNPs from 155 individuals. Node support values > 0.8 are shown as bootstrap from 1000 replicates. On the right, the species names are indicated in distinct colors. The scale bar represents genetic distance.

Among the *V. odorata*-like accession cluster, three genetic groups (GGs) were identified. GG 1, considered a genetically homogeneous group, consisted of 14 accessions from Mexico (ITTUX0005, ITTUX0006, ITTUX0013, ITTUX0014, ITTUX0015, ITTUX0016, ITTUX0017, ITTUX0019, ITTUX0022, ITTUX0024, ITTUX0026, ITTUX0027, ITTUX0030, and ITTUX0033) and two from Costa Rica (UNA-VAN-00245 and UNA-VAN-00246). GG 2 appeared as a hybrid resulting from a mixture of *V. odorata* and *V. insignis*/*V. cribbiana*, with a higher proportion (50–70%) of the *V. insignis*/*V. cribbiana* markers. This GG included one accession from Mexico (ITTUX0037), one accession from Costa Rica (UNA-VAN-00268), one accession from French Guyana (CR0116), and one accession from Brazil (CR3611). GG 3, similarly to GG 2, also appeared to be a hybrid between *V. odorata* and *V. insignis*/*V. cribbiana*, but with a lower proportion (25–30%) of the *V. insignis*/*V. cribbiana* markers. This GG included accessions from Mexico (ITTUX0055, ITTUX0066, and ITTUX0124). The identification of different genotypes within the *V. odorata*-like accessions is indicative of gene transfers between native vanilla populations, which supports genetic diversity while complicating the taxonomic identification of individuals. An unweighted Neighbor-Joining (NJ) tree was constructed from a genotyping matrix of 2365 SNPs across 155 individuals; it was generally congruent with the STRUCTURE analysis results, recovering three major genetic groups among the *V. odorata*-like accessions (Figure 3). The principal clusters corresponding to these groups showed moderate to high bootstrap support, although some internal relationships exhibited lower support values, suggesting unresolved relationships among closely related accessions. Therefore, additional phylogenetic studies will be necessary to further clarify the evolutionary relationships within the *V. odorata*-related group.

The three genotypes identified within the *V. odorata*-like accessions from Mexico corresponded to the variation observed in the morphological characterization. The *V. odorata* accession in GG 1 was characterized by small, broad leaves, flowers with the longest columns, reduced perianths, broad and elongated labella, and slender stems. In contrast, the *V. odorata* in GG 2 had large, narrow leaves, thick stems, and small fruits. Meanwhile, the *V. odorata* accession in GG 3 displayed larger, narrow leaves, slender stems, and large fruits.

## 3. Discussion

### 3.1. Morphological Variation and Taxonomic AFFINITIES WITHIn the Vanilla odorata Complex

The leaf morphology of the three Oaxaca odorata-like accessions is similar to that described for *V. odorata*: typically lanceolate leaves with a 7–23 cm × 1–3 cm acute apex [5,21,25,26]. Narrow and elongated leaves have also been observed in several species, most of which are considered conspecific to *V. odorata*, namely *V. uncinata*, *V. ensifolia*, *V. fimbriata*, *V. denticulata*, *Epidendrum vermifugum*, *V. labellopapillata*, and *V. karenchristianae* [5,25,26,27,28,34]. The leaves of the ITTUX0033 accession were the smallest, with morphological characteristics similar to those of *V. fimbriata* [25]. Soto-Arenas [26] and Chiron et al. [25] reported that *V. fimbriata* is characterized by similar but smaller leaves compared to *V. odorata*. This accession was collected in the same community (San Felipe, Usila, Oaxaca, Mexico) as the plant collected and described by Soto-Arenas [26], which the residents of that community call “Vainilla Tlatepusco”.

The stem morphologies of the Oaxaca odorata-like accessions are largely consistent with those of *V. odorata* from Mexico and Central America, i.e., terete, smooth, diameter of 0.4–0.6 cm, and internode length of 7–10.5 cm [21]. In contrast, these observations disagree with those reported by Soto-Arenas and Dressler [21] (diameter close to 0.8 cm, and internode length greater than 13.65 cm (Table 1)). The results also do not agree with the most recent taxonomic revision [5], where diameter values of 0.6 mm and internode lengths ranging from 6 to 12 cm are indicated. However, these vegetative characteristics are highly plastic and may vary depending on the local environmental conditions of the vines, which may contribute to the observed differences [13,14,15].

Floral morphology analysis provides key data for the discrimination and identification of related species. For example, it has been used to differentiate groups of leafless vanilla species from Madagascar [35,36] and to separate *Vanilla mexicana* Miller from *Vanilla ovata* Rolfe based on petal size [3]. Indeed, the characterization of the column, labellum, rostellum, and anther provides valuable insights into floral pollination. Species related to *V. odorata* are unlikely to self-pollinate due to the presence of a rostellum (Table 2), and flower dimensions may be indicative of the size and type of efficient pollinators.

The protologue of *V. odorata* described by Presl did not include a description of the flowers [25]. However, years later, some authors described the floral morphology of *V. odorata* from Mexico and Central America, reporting an adaxial sepal measuring 45–60 mm × 8–13 mm, lateral sepals with dimensions of 45–57 mm × 9–15 mm, petals with dimensions of 46–60 mm × 7–9 mm, and a labellum with dimensions of 41–55 mm × 22–25 mm [5,21,26]. Such a high degree of variation in a generally conserved trait (flowers) suggests that the historical descriptions of *V. odorata* may have included closely related species misidentified as *V. odorata*. The accessions from the three populations in Oaxaca exhibited contrasting morphological characteristics in their main floral parts, arguing in favor of distinct taxonomic positions.

The fruits of the species *V. odorata* range are 15–20 cm × 0.8–1 cm in size [5]. The fruit lengths of the studied accessions are within the reported range for the two main commercial species, *V. planifolia* (10–30 cm) [21] and *Vanilla ×tahitensis* J.W. Moore (10–19 cm) [37].

### 3.2. Genomic Evidence of Hybridization in Vanilla odorata-Related Taxa

Several genomics-based analyses have been conducted to assess the genetic diversity and species demarcation in the *Vanilla* Xanata section [6,7,24,38]. Currently, the reference genome for haplotype A CR0040, which corresponds to *V. planifolia*, represents the most comprehensive genomic resource available for the genus *Vanilla* [39]. Furthermore, the low fixation scores (FSTs) observed among closely related aromatic species, such as *V. planifolia*, *V. × tahitensis*, and *V. odorata*, suggest relatively close genomic relationships among these aromatic *Vanilla* taxa compared to more distantly related species within the genus [6].

The STRUCTURE analysis conducted in this study classified 155 accessions into four groups. One of these groups comprised accessions from the two species *V. cribbiana* and *V. insignis*. These two species are readily distinguished by their morphological characteristics, particularly the morphology of their flowers and stems. In addition, they differ in their aromatic properties: the fruits of *V. insignis* are non-aromatic, whereas the fruits of *V. cribbiana* are aromatic [40].

The *V. odorata*-like accessions were clustered in a group that exhibited three types of profiles, demonstrating significant genetic diversity between the populations from Oaxaca and the *V. odorata*-like accessions included in the STRUCTURE analysis. The accessions in GG 1 formed a pure group, but could hardly be assigned to *V. odorata* since their morphological characteristics—small and wide leaves—do not fit with the initial description of the species by Karol Presl [24]. However, Soto-Arenas [26] described *V. odorata* as a species with leaves measuring 8–13 cm × 1.5–2.7 cm, which aligns with the values obtained for GG 1 accessions. Additionally, GG 1 includes two accessions from Costa Rica (UNA-VAN-00245 and UNA-VAN-00246) where *V. odorata* has previously been reported [41,42].

GG 2 and GG 3 are considered hybrids as they exhibit a mixture of markers related to *V. odorata* and to *V. cribbiana* and/or *V. insignis*. The main difference is that GG 2 contains a higher percentage of the *V. insignis/V. cribbiana* markers (50–70%) compared to GG 3 (25–30%). In previous studies that used molecular markers (nuclear and plastid genes) to cluster accessions of the genus *Vanilla*, those related to *V. odorata* were clustered with *V. insignis*, which was identified as being closely related to *V. odorata* [22,24]. In the present study, the genomics-based technique (GBS) enabled the separation of *V. odorata*-related accessions from *V. insignis*. The GG 2 accession corresponds to Rancho Gavilán (Mexico; ITTUX0037) and is associated with hybrids from French Guiana (CR0116) and Brazil (CR3611). Additionally, the *V. odorata* GG 2 group appears to correspond to *V. ×tahitensis* accessions and hybrids from the Mexico and Réunion Island depositories (CR0003, CR0017, CR0097, CR1415, and ITTUX0083).

The accessions in the *V. odorata* GG 3 group originated from two localities: San Isidro, Naranjal, and Arroyo Choapam (Mexico; ITTUX0055, ITTUX0066, and ITTUX0124). Given that these localities are only 10 km apart, it is possible that they share the same origin. However, to date, the ITTUX0055 accession has not flowered, preventing its morphological characterization, which is necessary to determine if there are differences between the accessions from these localities. *V. odorata* GG 3 only includes accessions from Mexico.

Although all accessions were maintained under homogeneous ex situ conditions in the germplasm bank, clear morphological differences between populations were consistently observed. This suggests that the detected variation is unlikely to result solely from environmental plasticity and may instead reflect underlying genetic differences. Notably, the three populations originated from habitats differing in humidity, canopy cover, and environmental exposure, factors that may contribute to ecological divergence and local adaptation of the *V. odorata*-like complex.

Together with the phylogenetic and admixture patterns observed in this study, these findings support the hypothesis that diversification among odorata-like *Vanilla* populations may involve ecological differentiation and hybridization among closely related *Vanilla* taxa.

## 4. Materials and Methods

### 4.1. Collection and Preservation of Vegetative Material

**Figure 4:**
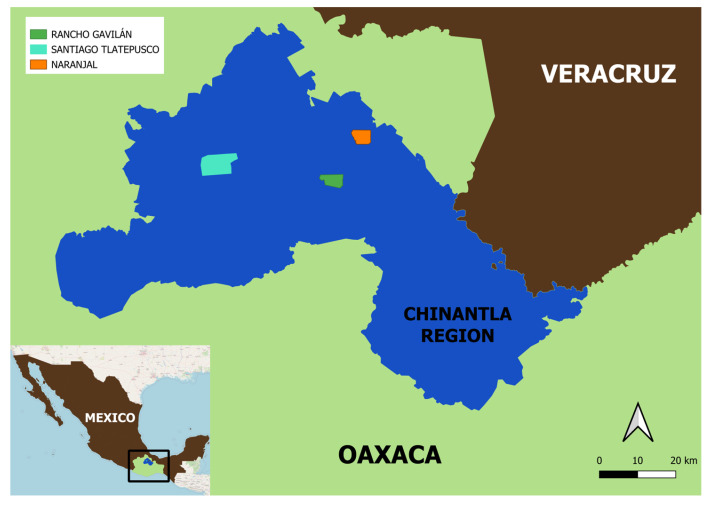
Locations of *V. odorata*-like populations collected in Mexico. Chinantla region (blue), Oaxaca, Mexico (light green) and Veracruz (brown).

Field surveys were conducted in three localities within the Chinantla region of Oaxaca, Mexico, where wild populations of the genus *Vanilla* that exhibit morphological traits similar to *Vanilla odorata* had previously been reported and identified by Soto Arenas [26]. During these surveys, plant specimens were collected under permit SPARN/DGVS/12324/23 issued by the Secretaría de Medio Ambiente y Recursos Naturales (SEMARNAT). Plant material was collected from Santiago Tlatepusco (ITTUX0033), Rancho Gavilán (ITTUX0037), and San Isidro Naranjal (ITTUX0066) (Figure 4). The collected specimens were preserved as live specimens at the *Vanilla* conservation bank of the Tecnológico Nacional de México’s Tuxtepec campus (TNM/ITTUX). In addition, dehydrated specimens were sent to the National Herbarium of Mexico (MEXU), Institute of Biology, UNAM (accession numbers: 1592843 (ITTUX0033), 1592844 (ITTUX0037), and 1592842 (ITTUX0066)).

**Figure 5:**
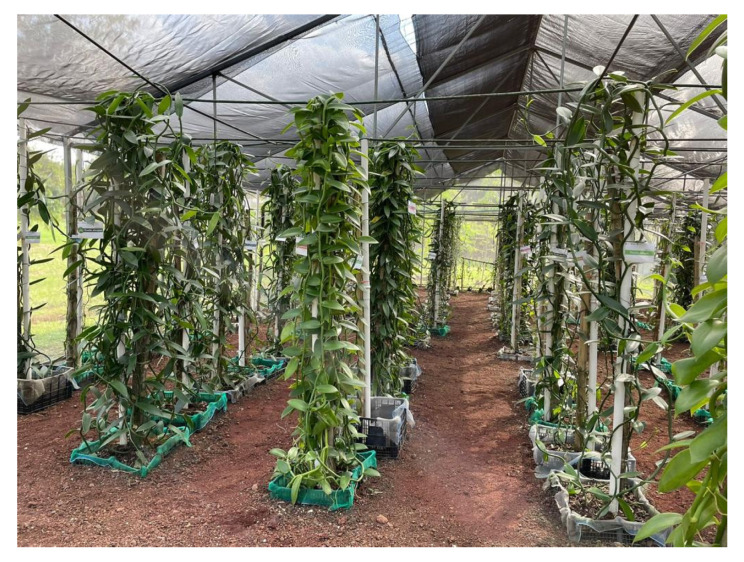
Vanilla conservation bank at Tuxtepec campus of Tecnológico Nacional de México.

**Figure 6:**
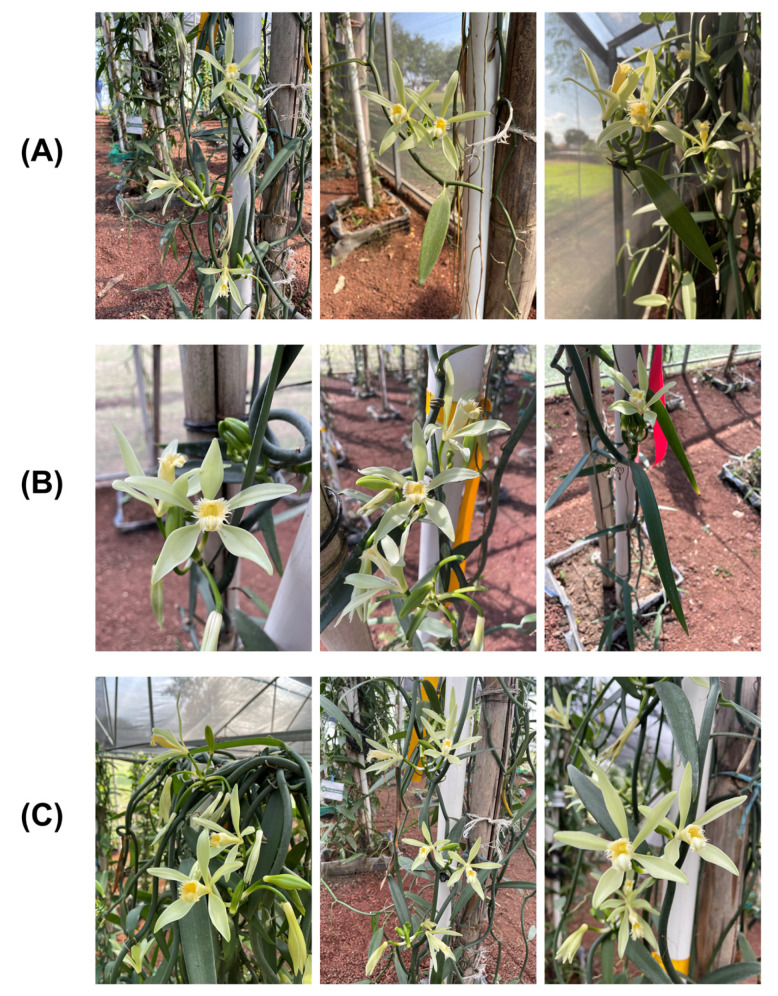
Flowering of different accessions related to *V. odorata* from the Chinantla region, Oaxaca, Mexico: (**A**) ITTUX0033, (**B**) ITTUX0037, and (**C**) ITTUX0066.

The conservation bank is located in San Juan Bautista, Tuxtepec, Oaxaca, and comprises two 200 m^2^ plots equipped with a 50% shade mesh structure. All *Vanilla odorata* plants were grown in plastic containers with PVC pipes serving as vertical supports and were maintained under uniform climatic and agronomic management conditions (Figure 5). The average climatic conditions were as follows: minimum and maximum temperatures ranged from 18.7 to 35.8 °C, and relative humidity varied between 71.8% and 88.1%. A sprinkler irrigation system was primarily used during dry seasons to maintain a minimum relative humidity of 80%. Plant nutrition consisted of a 1:1 mixture of coconut fiber and EcoSustrato^®^ orchid substrate (PISUMMA, Ciudad de México, Mexico). After two years of growth under ex situ conservation conditions, the first flowering occurred, and the flowers were manually pollinated (Figure 6). At nine months after pollination, when the fruits began to show slight yellow coloration, the fruits were harvested.

### 4.2. Vegetative Material and Morphological Analysis

The morphological study of the accessions related to *Vanilla odorata* was conducted exclusively using material from Mexico as these accessions were maintained under uniform conditions in the germplasm bank. Reproductive vegetative materials (fruiting specimens) from other regions, such as Costa Rica and France, were not available for analysis. Therefore, the morphological assessment was restricted to accessions originating from Mexico.

For morphological characterization, ten measurements were taken for each plant part of the Oaxaca odorata-like accessions. Leaves, stems, and fruits were analyzed using both qualitative and quantitative descriptors following the guidelines established by the UPOV [16] for *Vanilla*. The qualitative descriptors included shape, apex shape, and base shape for leaves; shape and surface for stems; and shape and cross-sectional shape for fruits. Quantitative descriptors were measured using a balance and a digital caliper; these included leaf length and width; stem diameter and internode length; and fruit length, width, and weight.

**Figure 7:**
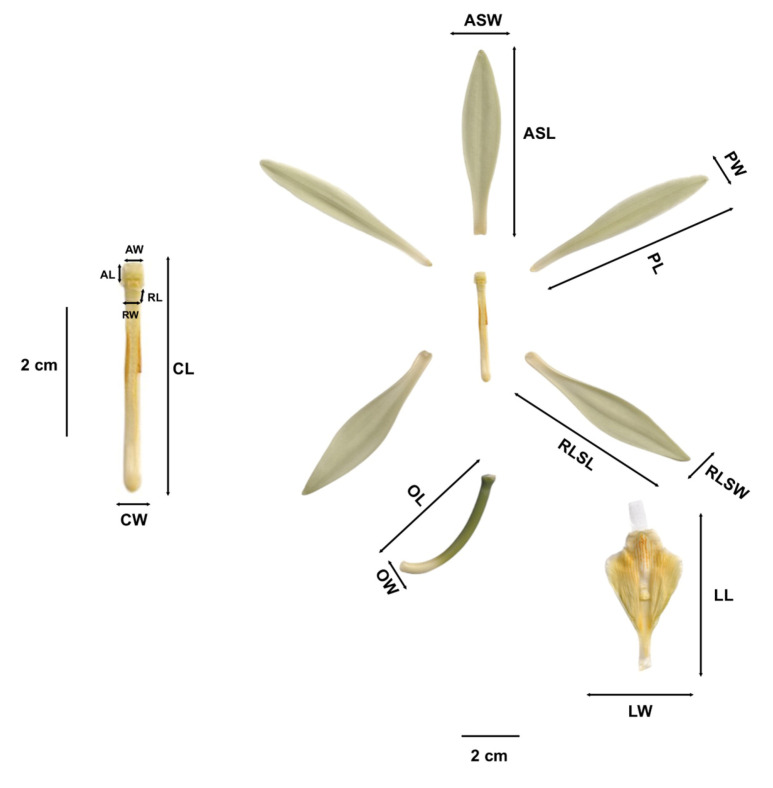
Descriptors evaluated in flowers of different accessions related to *V. odorata*: ovary length (OL), ovary width (OW), adaxial sepal length (ASL), adaxial sepal width (ASW), right lateral sepal length (RLSL), right lateral sepal width (RLSW), petal length (PL), petal width (PW), labellum length (LL), labellum width (LW), column length (CL), column width (CW), rostellum length (RL), rostellum width (RW), anther length (AL), and anther width (AW).

For flowers, the methodology reported by Andriamihaja et al. [36] was followed. Ten flowers were dissected, and the following descriptors were measured: flower weight (FW), ovary weight (OWE), ovary length (OL), ovary width (OW), adaxial sepal length (ASL), adaxial sepal width (ASW), right lateral sepal length (RLSL), right lateral sepal width (RLSW), petal length (PL), petal width (PW), labellum length (LL), labellum width (LW), column length (CL), column width (CW), rostellum length (RL), rostellum width (RW), anther length (AL), and anther width (AW) (Figure 7).

### 4.3. Comparative Morphometric Analysis

The morphometric values of the leaves, stems, and flowers from the Oaxaca odorata-like accessions were compared with values reported in the literature for odorata-like species. The comparative dataset was obtained from Chiron et al. [25], who analyzed photographs of vegetative material from herbarium specimens of species related to *V. odorata*, and from field data collected during botanical expeditions conducted in Costa Rica through Universidad Nacional de Costa Rica [41,43]. While we acknowledge that dehydration of herbarium specimens can induce tissue shrinkage (potentially affecting absolute measurements of leaves and stems), this limitation was mitigated by prioritizing morphological ratios (e.g., length/width) and diagnostic floral traits, which remain relatively consistent regardless of the preservation method.

### 4.4. Material for Genetic Analysis

For the phylogenetic study, in addition to *V. odorata* accessions and other *Vanilla* species from Mexico, accessions from Costa Rica and the Vatel Biological Resource Center (BRC), Saint Pierre, Réunion Island, France, were included (Table 3).

### 4.5. Genetic Analysis

The genetic analysis was carried out on 155 accessions from different collections—from Mexico, Costa Rica, and Réunion Island. High-molecular-weight DNA was extracted from 25 mg of lyophilized leaves for each accession using the DNeasy Plant Mini Kit (Qiagen, Hilden, Germany). DNA quantification was performed using a Qubit 2.0 fluorometer (Thermo Fisher Scientific, Waltham, MA, USA) and concentrations were normalized to 50 ng/µL. DNA integrity and quality were assessed using electrophoresis on a 2% agarose gel.

Library preparation was carried out at the Regional Genotyping Technology Platform (UMR AGAP, CIRAD, Montpellier, France) following the protocol described by Elshire et al. [44]. Genomic DNA was digested using the PstI restriction enzyme (New England Biolabs, Ipswich, MA, USA) and libraries were sequenced on a HiSeq3000 sequencer (Illumina Inc., San Diego, CA, USA) using a paired-end sequencing approach at the GeT-PlaGe platform (INRAE, Toulouse, France).

The quality of the 150 bp paired-end raw reads was assessed using FastQC (v0.11.7; https://www.bioinformatics.babraham.ac.uk/projects/fastqc/; accessed on 11 May 2024). Reads containing Illumina adapter sequences and overrepresented sequences were removed using Cutadapt (v3.5, Martin [45]). The demultiplexing of reads was performed using the GBS barcode splitter tool (https://sourceforge.net/projects/gbsbarcode/; accessed on 28 May 2024), and sequences were trimmed to 142 bp to standardize read lengths across accessions. Samples with fewer than 100,000 reads were excluded from the analysis, and reads from replicates were concatenated.

SNP calling was performed using both de novo and reference-based approaches with the STACKS pipeline [46]. For the reference-based approach, reads were aligned to the CR0040 Haplotype A reference genome [39] using BWA aligner (v0.7.17, Li and Durbin [47]). SNPs with more than 30% missing data and a minor allele frequency below 10% were discarded. The remaining SNPs were converted into a Variant Call Format (VCF; Danecek et al. [48]) file and visualized using the vcfR package (v1.12.0, Knaus and Grünwald [49], R Development Core Team 2010). A maximum of three SNPs per locus were selected for genetic analysis.

Population structure was inferred using the Bayesian clustering method implemented in STRUCTURE (v2.3.4, Pritchard et al. [50]) to identify clusters of genetically related individuals. The admixture model was applied. For each K value (ranging from K = 1 to K = 8), three independent runs were performed, each consisting of a burn-in period of 10,000 iterations followed by 100,000 Markov Chain Monte Carlo (MCMC) iterations. The optimal K was determined using the ΔK method (Evanno et al. [33]) by running STRUCTURE HARVESTER (Earl & vonHoldt [51]).

From the complete genotyping dataset, a dissimilarity coefficient was calculated using DarWIN software [52] and the simple matching index [53]. Distance trees were constructed from 1000 bootstrap replicates using the Unweighted Neighbor-Joining method [54]. Trees were then converted into Phylip files and plotted using FigTree software, v1.4.4 [55].

### 4.6. Statistical Analysis

The values obtained from the accessions were compared using one-way ANOVA. Significant differences were determined using Tukey’s test (*p* ≤ 0.05) and MINITAB software, v17.

## 5. Conclusions

The application of dual taxonomy approaches (morphology and genomics) enabled the differentiation and identification of three congruent groups within wild populations related to *V. odorata* from various localities in the state of Oaxaca, Mexico. The observed morphological and genomic variation highlights the considerable diversity present within aromatic wild vanilla populations and supports the existence of complex relationships among closely related taxa within the *V. odorata* group.

The Genotyping-by-Sequencing analyses revealed a genetically homogeneous lineage represented by the GG 1 accessions, which may correspond to a previously unrecognized taxonomic entity. However, additional studies incorporating expanded morphological sampling, reproductive analyses, and formal taxonomic evaluation will be necessary to conclusively determine its status. In contrast, the GG 2 and GG 3 accessions exhibited genomic admixture patterns consistent with hybridization involving lineages related to *V. odorata*, *V. insignis*, and/or *V. cribbiana*.

The results of this study also support the hypothesis that the current taxonomic circumscription and typification framework for *V. odorata* may require reassessment, potentially involving materials of hybrid origin. Consequently, a comprehensive nomenclatural revision of the *V. odorata* complex, including the evaluation of lectotypes, neotypes, and epitypes, is strongly recommended for future research.

## Figures and Tables

**Table 1:** Morphological characteristics of leaves, stems, flowers, and fruits from Oaxaca odorata-like accessions. Values are presented as the mean ± standard deviation (SD).

Part of Plant ^†^	Descriptors	Accessions		
		ITTUX0033	ITTUX0037	ITTUX0066
**Leaf**	Length (cm)	14.94 ± 0.56 ^c^	19.96 ± 0.87 ^a^	19.06 ± 0.84 ^b^
	Width (cm)	2.43 ± 0.09 ^a^	2.28 ± 0.19 ^ab^	2.16 ± 0.17 ^b^
	Shape	Lanceolate	Lanceolate	Lanceolate
	Base	Acute	Attenuate	Attenuate
	Apex	Obtuse	Acute	Acute
	L/W ratio (cm)	6.16 ± 0.34 ^b^	8.81 ± 0.62 ^a^	8.85 ± 0.56 ^a^
**Stem**	Shape	Terete	Terete	Terete
	Diameter (cm)	0.76 ± 0.02 ^b^	0.84 ± 0.07 ^a^	0.79 ± 0.03 ^b^
	Internode length (cm)	15 ± 0.82 ^b^	13.65 ± 0.83 ^c^	18.02 ± 0.84 ^a^
	Surface	Smooth	Smooth	Smooth
**Flower**	Flower weight (g) (FW)	1.64 ± 0.24 ^b^	1.74 ± 0.07 ^ab^	2.04 ± 0.25 ^a^
	Ovary weight (g) (OWE)	0.25 ± 0.06 ^b^	0.33 ± 0.03 ^ab^	0.37 ± 0.07 ^a^
	Ovary length (mm) (OL)	34.41 ± 2.44 ^b^	36.60 ± 2.94 ^ab^	41.31 ± 1.92 ^a^
	Ovary width (mm) (OW)	3.58 ± 0.14 ^a^	3.77 ± 0.34 ^a^	3.79 ± 0.49 ^a^
	Column length (mm) (CL)	37.88 ± 1.79 ^a^	33.09 ± 0.60 ^c^	35.94 ± 0.78 ^b^
	Column width (mm) (CW)	2.55 ± 0.09 ^a^	2.57 ± 0.24 ^a^	2.75 ± 0.36 ^a^
	Labellum length (mm) (LL)	49.10 ± 1.95 ^a^	43.00 ± 0.29 ^c^	47.62 ± 0.75 ^b^
	Labellum width (mm) (LW)	20.70 ± 0.69 ^c^	21.81 ± 0.38 ^b^	24.44 ± 0.62 ^a^
	Adaxial sepal length (mm) (ASL)	56.72 ± 3.39 ^a^	56.49 ± 0.99 ^a^	59.18 ± 1.74 ^a^
	Adaxial sepal width (mm) (ASW)	9.47 ± 1.21 ^c^	10.92 ± 0.43 ^b^	12.67 ± 0.87 ^a^
	Right lateral sepal length (mm) (RLSL)	55.88 ± 3.47 ^a^	54.88 ± 1.70 ^a^	56.92 ± 2.30 ^a^
	Right lateral sepal width (mm) (RLSW)	11.30 ± 1.89 ^b^	12.68 ± 0.58 ^a^	12.75 ± 1.30 ^a^
	Petal length (mm) (PL)	55.81 ± 3.13 ^a^	54.63 ± 1.65 ^a^	57.44 ± 1.71 ^a^
	Petal width (mm) (PW)	7.59 ± 1.51 ^b^	8.16 ± 0.86 ^ab^	9.02 ± 1.06 ^a^
	Rostellum length (mm) (RL)	2.17 ± 0.05 ^b^	2.81 ± 0.15 ^a^	2.63 ± 0.29 ^a^
	Rostellum width (mm) (RW)	2.78 ± 0.14 ^a^	2.77 ± 0.11 ^a^	2.68 ± 0.31 ^a^
	Anther length (mm) (AL)	2.84 ± 0.25 ^a^	2.75 ± 0.21 ^a^	2.69 ± 0.21 ^a^
	Anther width (mm) (AW)	2.77 ± 0.13 ^a^	2.67 ± 0.13 ^a^	2.71 ± 0.14 ^a^
**Fruit**	Shape	Oblong	Oblong	Oblong
	Shape in cross-section	Triangular	Triangular	Triangular
	Length (cm)	18.92 ± 0.61 ^a^	15.66 ± 1.36 ^b^	19.68 ± 0.52 ^a^
	Width (mm)	11.64 ± 0.40 ^ab^	11.30 ± 0.77 ^b^	12.11 ± 0.54 ^a^
	Weight (g)	9.00 ± 1.23 ^b^	8.20 ± 1.58 ^b^	10.97 ± 0.57 ^a^

^†^ Ten samples of the vegetative parts were analyzed. Values with different letters between columns are significantly different according to Tukey’s test at a significance level of 0.05.

**Table 2:** Comparison of morphological characteristics of leaves, stems, and flowers of the studied accessions and species related to *V. odorata*.

Descriptors (cm)	Samples			*V.* *odorata* ^†^	Species Related to *V. odorata*		
	ITTUX0066	ITTUX0033	ITTUX0037		*V.* *ensifolia* ^†^	*V.* *fimbriata* ^†^	*V.* *karenchristianae* ^‡^
**Leaves** **(L/W)**	8.90 ± 0.56	6.16 ± 0.34	8.81 ± 0.62	≥10	~8–10	~5–6.5	7.2 ± 1.24
**Dorsal sepal** **(L/W)**	4.71 ± 0.33	6.03 ± 0.53	5.18 ± 0.22	~4.5	~8	~7	5.5 ± 0.34
**Relative** I**L/L**	0.95 ± 0.07	1.00 ± 0.05	0.68 ± 0.05	~0.5–0.7	~0.7	~1–1.5	0.91 ± 0.01

L/W: leaf length/width ratio; IL/L: internode length/leaf ratio. ^†^ Chiron et al. [25]. ^‡^ From field data of the collecting missions in Costa Rica.

**Table 3:** Identification and origin of the accessions of *Vanilla* spp. used in this study.

Accession Number	*Vanilla* Species	Geographic Origin	Depository
ITTUX0005	*V. odorata* cf.	Santiago Tlatepusco, Oaxaca, Mexico	TNM/ITTUX
ITTUX0006	*V. odorata* cf.	Santiago Tlatepusco, Oaxaca, Mexico	TNM/ITTUX
ITTUX0013	*V. odorata* cf.	Santiago Tlatepusco, Oaxaca, Mexico	TNM/ITTUX
ITTUX0014	*V. odorata* cf.	Santiago Tlatepusco, Oaxaca, Mexico	TNM/ITTUX
ITTUX0015	*V. odorata* cf.	Santiago Tlatepusco, Oaxaca, Mexico	TNM/ITTUX
ITTUX0016	*V. odorata* cf.	Santiago Tlatepusco, Oaxaca, Mexico	TNM/ITTUX
ITTUX0017	*V. odorata* cf.	Santiago Tlatepusco, Oaxaca, Mexico	TNM/ITTUX
ITTUX0019	*V. odorata* cf.	Santiago Tlatepusco, Oaxaca, Mexico	TNM/ITTUX
ITTUX0022	*V. odorata* cf.	Santiago Tlatepusco, Oaxaca, Mexico	TNM/ITTUX
ITTUX0024	*V. odorata* cf.	Santiago Tlatepusco, Oaxaca, Mexico	TNM/ITTUX
ITTUX0026	*V. odorata* cf.	Santiago Tlatepusco, Oaxaca, Mexico	TNM/ITTUX
ITTUX0027	*V. odorata* cf.	Santiago Tlatepusco, Oaxaca, Mexico	TNM/ITTUX
ITTUX0028	*V. planifolia*	Region Mixe, Oaxaca, Mexico	TNM/ITTUX
ITTUX0030	*V. odorata* cf.	Santiago Tlatepusco, Oaxaca, Mexico	TNM/ITTUX
ITTUX0033	*V. odorata* cf.	Santiago Tlatepusco, Oaxaca, Mexico	TNM/ITTUX
ITTUX0034	*V. planifolia*	Region Mixe, Oaxaca, Mexico	TNM/ITTUX
ITTUX0035	*V. planifolia*	Region Mixe, Oaxaca, Mexico	TNM/ITTUX
ITTUX0037	*V. odorata* cf.	Rancho Gavilán, Oaxaca, Mexico	TNM/ITTUX
ITTUX0038	*V.* sp	Chajul, Chiapas, Mexico	TNM/ITTUX
ITTUX0039	*V. planifolia*	Pueblo Nuevo, Oaxaca, Mexico	TNM/ITTUX
ITTUX0040	*V. planifolia*	Loma San Rafael, Oaxaca, Mexico	TNM/ITTUX
ITTUX0041	*V. planifolia*	Pueblo Nuevo, Oaxaca, Mexico	TNM/ITTUX
ITTUX0042	*V. insignis*	Mazin Grande, Oaxaca, Mexico	TNM/ITTUX
ITTUX0044	*V. pompona*	Emiliano Zapata, Oaxaca, Mexico	TNM/ITTUX
ITTUX0045	*V. pompona*	Tuxtepec, Oaxaca, Mexico	TNM/ITTUX
ITTUX0046	*V. pompona*	Guerrero, Mexico	TNM/ITTUX
ITTUX0047	*V. cribbiana*	Cobán, Guatemala	TNM/ITTUX
ITTUX0048	*V. planifolia*	Loma San Rafael, Oaxaca	TNM/ITTUX
ITTUX0049	*V. insignis*	Xalapa, Veracruz, Mexico	TNM/ITTUX
ITTUX0050	*V. pompona*	Cerro Tepezcuintle, Oaxaca, Mexico	TNM/ITTUX
ITTUX0051	*V. planifolia*	San Isidro Naranjal, Oaxaca, Mexico	TNM/ITTUX
ITTUX0054	*V. pompona*	Tuxtepec, Oaxaca, Mexico	TNM/ITTUX
ITTUX0055	*V. odorata* cf.	Arroyo Choapam, Oaxaca, Mexico	TNM/ITTUX
ITTUX0056	*V. insignis*	Mazin Grande, Oaxaca, Mexico	TNM/ITTUX
ITTUX0057	*V. insignis*	Xalapa, Veracruz, Mexico	TNM/ITTUX
ITTUX0059	*V.* sp	Rancho Gavilán, Oaxaca, Mexico	TNM/ITTUX
ITTUX0060	*V.* sp	Rancho Gavilán, Oaxaca, Mexico	TNM/ITTUX
ITTUX0062	*V. insignis*	Xalapa, Veracruz, Mexico	TNM/ITTUX
ITTUX0064	*V. pompona*	Ayautla Oaxaca, Mexico	TNM/ITTUX
ITTUX0065	*V. planifolia*	San Bartolome Ayautla Oaxaca, Mexico	TNM/ITTUX
ITTUX0066	*V. odorata* cf.	San Isidro Naranjal, Oaxaca, Mexico	TNM/ITTUX
ITTUX0070	*V. planifolia*	Papantla, Veracruz, Mexico	TNM/ITTUX
ITTUX0072	*V.* sp	Mexico	TNM/ITTUX
ITTUX0073	*V.* sp	Mexico	TNM/ITTUX
ITTUX0074	*V.* sp	Mexico	TNM/ITTUX
ITTUX0075	*V. pompona*	Xalapa, Veracruz, Mexico	TNM/ITTUX
ITTUX0077	*V. pompona*	Tuxtepec, Oaxaca, Mexico	TNM/ITTUX
ITTUX0080	*V. planifolia*	San Bartolome Ayautla, Oaxaca, Mexico	TNM/ITTUX
ITTUX0081	*V. pompona*	María Lombardo, Oaxaca, Mexico	TNM/ITTUX
ITTUX0082	*V. pompona*	Cerro Tepezcuintle, Oaxaca, Mexico	TNM/ITTUX
ITTUX0083	*V.* x *tahitensis*	Guatemala	TNM/ITTUX
ITTUX0084	*V.pompona*	Cerro Armadillo, Oaxaca, Mexico	TNM/ITTUX
ITTUX0085	*V. pompona*	Loma San Rafael, Oaxaca, Mexico	TNM/ITTUX
ITTUX0087	*V. pompona*	Loma San Rafael, Oaxaca, Mexico	TNM/ITTUX
ITTUX0088	*V. cribbiana*	Cobán, Guatemala	TNM/ITTUX
ITTUX0089	*V. pompona*	Nayarit, Mexico	TNM/ITTUX
ITTUX0090	*V. pompona*	Nayarit, Mexico	TNM/ITTUX
ITTUX0092	*V. pompona*	Mixe, Oaxaca, Mexico	TNM/ITTUX
ITTUX0093	*V. pompona*	Chimalapas, Oaxaca, Mexico	TNM/ITTUX
ITTUX0094	*V. pompona*	Veracruz, Mexico	TNM/ITTUX
ITTUX0095	*V. planifolia*	Veracruz, Mexico	TNM/ITTUX
ITTUX0096	*V. planifolia*	Veracruz, Mexico	TNM/ITTUX
ITTUX0097	*V. planifolia*	Zongolica, Veracruz, Mexico	TNM/ITTUX
ITTUX0099	*V. planifolia*	Veracruz, Mexico	TNM/ITTUX
ITTUX0100	*V. planifolia*	Veracruz, Mexico	TNM/ITTUX
ITTUX0101	*V. pompona*	María Lombardo, Oaxaca, Mexico	TNM/ITTUX
ITTUX0104	*V. planifolia*	Valle Nacional, Oaxaca, Mexico	TNM/ITTUX
ITTUX0105	*V. planifolia*	Valle Nacional, Oaxaca, Mexico	TNM/ITTUX
ITTUX0114	*V.* sp	Naha, Chiapas, Mexico	TNM/ITTUX
ITTUX0115	*V.* sp	Metzabok, Chiapas, Mexico	TNM/ITTUX
ITTUX0117	*V. planifolia*	Santa Maria Lachichina, Oaxaca, Mexico	TNM/ITTUX
ITTUX0118	*V. planifolia*	La Lopa, Oaxaca, Mexico	TNM/ITTUX
ITTUX0119	*V. planifolia*	La Lopa, Oaxaca, Mexico	TNM/ITTUX
ITTUX0120	*V. planifolia*	Reagui, Oaxaca, Mexico	TNM/ITTUX
ITTUX0121	*V. planifolia*	Reagui, Oaxaca, Mexico	TNM/ITTUX
ITTUX0122	*V. insignis*	San Isidro Naranjal, Oaxaca, Mexico	TNM/ITTUX
ITTUX0123	*V. insignis*	San Isidro Naranjal, Oaxaca, Mexico	TNM/ITTUX
ITTUX0124	*V. odorata* cf.	San Isidro Naranjal, Oaxaca, Mexico	TNM/ITTUX
ITTUX0125	*V.* sp	Naha, Chiapas, Mexico	TNM/ITTUX
ITTUX0130	*V.* sp	Tres Lagunas, Chiapas, Mexico	TNM/ITTUX
ITTUX0133	*V.* sp	Arroyo Choapam, Oaxaca, Mexico	TNM/ITTUX
ITTUX0134	*V. planifolia*	Santa María Chilchotla, Oaxaca, Mexico	TNM/ITTUX
ITTUX0139	*V. pompona*	Pueblo Nuevo Ojo de Agua, Oaxaca, Mexico	TNM/ITTUX
ITTUX0140	*V. pompona*	Pueblo Nuevo Ojo de Agua, Oaxaca, Mexico	TNM/ITTUX
ITTUX0146	*V. planifolia*	Papantla, Veracruz, Mexico	TNM/ITTUX
ITTUX0151	*V.* sp	San Jose Tenango, Oaxaca, Mexico	TNM/ITTUX
ITTUX0159	*V.* sp	San Andres Tuxtla, Veracruz, Mexico	TNM/ITTUX
ITTUX0161	*V. planifolia*	Papantla, Veracruz, Mexico	TNM/ITTUX
ITTUX0168	*V. pompona*	Santa Cruz Itundujia, Oaxaca, Mexico	TNM/ITTUX
ITTUX0173	*V. pompona*	María Lombardo, Oaxaca, Mexico	TNM/ITTUX
ITTUX0176	*V. planifolia*	Papantla, Veracruz, Mexico	TNM/ITTUX
ITTUX0180	*V.* sp	Guatemala	TNM/ITTUX
ITTUX0181	*V.* sp	Guatemala	TNM/ITTUX
ITTUX0182	*V.* sp	Guatemala	TNM/ITTUX
ITTUX0183	*V.* sp	Guatemala	TNM/ITTUX
ITTUX0184	*V. planifolia*	Gutiérrez Zamora, Veracruz, Mexico	TNM/ITTUX
ITTUX0185	*V. planifolia*	Gutiérrez Zamora, Veracruz, Mexico	TNM/ITTUX
CR0001	*V. planifolia*	Reunion Island, France	BRC VATEL
CR0003	*V. planifolia* x *V.* x *tahitensis*	Madagascar	BRC VATEL
CR0017	*V.* x *tahitensis*	French Polynesia	BRC VATEL
CR0018	*V. pompona*	French Polynesia	BRC VATEL
CR0020	*V. planifolia*	BRC Vatel, CIRAD, France	BRC VATEL
CR0033	*V. pompona*	Reunion Island, France	BRC VATEL
CR0038	*V. planifolia*	Reunion Island, France	BRC VATEL
CR0040	*V. planifolia*	Reunion Island, France	BRC VATEL
CR0046	*V. pompona*	Guadeloupe, France	BRC VATEL
CR0068	*V. sotoarenasii*	Cahuita, Costa Rica	BRC VATEL
CR0069	*V. pompona*	Private collection, France	BRC VATEL
CR0070	*V. pompona*	Private collection, France	BRC VATEL
CR0072	*V. bahiana*	Bahia, Brazil	BRC VATEL
CR0093	*V. pompona*	Private collection, France	BRC VATEL
CR0096	*V. pompona*	Private collection, France	BRC VATEL
CR0097	*V. bahiana*	Bahia, Brazil	BRC VATEL
CR0109	*V. cribbiana*	Private collection, France	BRC VATEL
CR0116	*V. odorata* cf.	French Guyana, France	BRC VATEL
CR0126	*V. planifolia*	Antalaha, Madagascar	BRC VATEL
CR0139	*V. planifolia* x *V. pompona* hybrid	Madagascar	BRC VATEL
CR0626	*V. planifolia*	Reunion Island, France	BRC VATEL
CR0628	*V. planifolia*	Reunion Island, France	BRC VATEL
CR0630	*V. planifolia*	Reunion Island, France	BRC VATEL
CR0641	*V. planifolia*	Reunion Island, France	BRC VATEL
CR0693	*V. pompona*	Private collection, France	BRC VATEL
CR1156	*V.sotoarenasii* x *V. pompona* hybrid hybrid	BRC Vatel, CIRAD, France	BRC VATEL
CR1415	*V.* x *tahitensis*	BRC Vatel, CIRAD, France	BRC VATEL
CR1433	*V. planifolia*	BRC Vatel, CIRAD, France	BRC VATEL
CR1725	*V. planifolia x V. pompona* hybrid	BRC Vatel, CIRAD, France	BRC VATEL
CR1900	*V. planifolia*	BRC Vatel, CIRAD, France	BRC VATEL
CR2527	*V. planifolia*	BRC Vatel, CIRAD, France	BRC VATEL
CR2688	*V. insignis*	Taurino Tràpaga mantano, Mexico	BRC VATEL
CR2717	(*V. planifolia x V. pompona) x V. planifolia*	Costa Rica	BRC VATEL
CR2718	(*V. planifolia* x *V. pompona) x V. planifolia*	Costa Rica	BRC VATEL
CR2897	*V. cribbiana*	Tena, Ecuador	BRC VATEL
CR3611	*V. odorata* cf.	Ilheus, Bahia, Brazil	BRC VATEL
UNA-VAN-00245	*V. odorata* cf.	Pacuarito, Limón, Costa Rica	UNA
UNA-VAN-00246	*V. odorata* cf.	Pacuarito, Limón, Costa Rica	UNA
UNA-VAN-00247	*V. pompona*	Pococí, Limón, Costa Rica	UNA
UNA-VAN-00248	Costa Rica hybrid	Pococí, Limón, Costa Rica	UNA
UNA-VAN-00249	Costa Rica hybrid	Pococí, Limón, Costa Rica	UNA
UNA-VAN-00250	Costa Rica hybrid	Pococí, Limón, Costa Rica	UNA
UNA-VAN-00251	*V.* sp	Pérez Zeledón, San José, Costa Rica	UNA
UNA-VAN-00252	*V. phaeantha*	Buenos Aires, Puntarenas, Costa Rica	UNA
UNA-VAN-00253	*V.* sp	Guácimo, Limón, Costa Rica	UNA
UNA-VAN-00254	*V. karenchristianae*	Siquirres, Limón, Costa Rica	UNA
UNA-VAN-00255	*V. karenchristianae*	Siquirres, Limón, Costa Rica	UNA
UNA-VAN-00259	*V. dressleri*	Siquirres, Limón, Costa Rica	UNA
UNA-VAN-00260	*V.* sp	Los Chiles, Alajuela, Costa Rica	UNA
UNA-VAN-00261	*V.* sp	Los Chiles, Alajuela, Costa Rica	UNA
UNA-VAN-00263	*V.* sp	Puerto Jiménez, Puntarenas, Costa Rica	UNA
UNA-VAN-00264	*V. pompona*	Puerto Jiménez, Puntarenas, Costa Rica	UNA
UNA-VAN-00265	*V. trigonocarpa*	Puerto Jiménez, Puntarenas, Costa Rica	UNA
UNA-VAN-00266	*V.* sp	Puerto Jiménez, Puntarenas, Costa Rica	UNA
UNA-VAN-00268	*V.* sp	Puerto Jiménez, Puntarenas, Costa Rica	UNA
UNA-VAN-00269	*V.* sp	Puerto Jiménez, Puntarenas, Costa Rica	UNA
UNA-VAN-00272	*V.* sp	Osa, Puntarenas, Costa Rica	UNA
UNA-VAN-00273	*V.* sp	Osa, Puntarenas, Costa Rica	UNA

## Data Availability

Biological samples used in this study have been deposited in the BioSamples Database under the accession identifier SAMEA118635983-SAMEA118636137.

## Supplementary Materials

The following supporting information can be downloaded at: https://www.mdpi.com/article/10.3390/plants15111661/s1, Figure S1: Morphological datasheet of accession ITTUX0033. (A) Stem, (B) Leaf, (C) Fruit, (D) Fruit in cross-section, (E) Flower, (F) Sepals and petals, (G) Ovary, (H) Column and (I) Labellum. Figure S2: Morphological datasheet of accession ITTUX0037. (A) Stem, (B) Leaf, (C) Fruit, (D) Fruit in cross-section, (E) Flower, (F) Sepals and petals, (G) Ovary, (H) Column and (I) Labellum. Figure S3: Morphological datasheet of accession ITTUX0066. (A) Stem, (B) Leaf, (C) Fruit, (D) Fruit in cross-section, (E) Flower, (F) Sepals and petals, (G) Ovary, (H) Column and I) Labellum. Figure S4: DeltaK graph shows the best K as determined by STRUCTURE HARVESTER.
