# Supplementary material for: Genetic and Morphological Analyses of Native Vanilla Populations in Mexico Call into Question the Taxonomy of V. odorata
Source: Plants (Basel). 2026 May 28;15(11):1661. doi: 10.3390/plants15111661 (PMC13258980; doi:10.3390/plants15111661)
Supplement: Supplementary file 1 [file plants-15-01661-s001.zip › plants-4301467-supplementary.pdf]

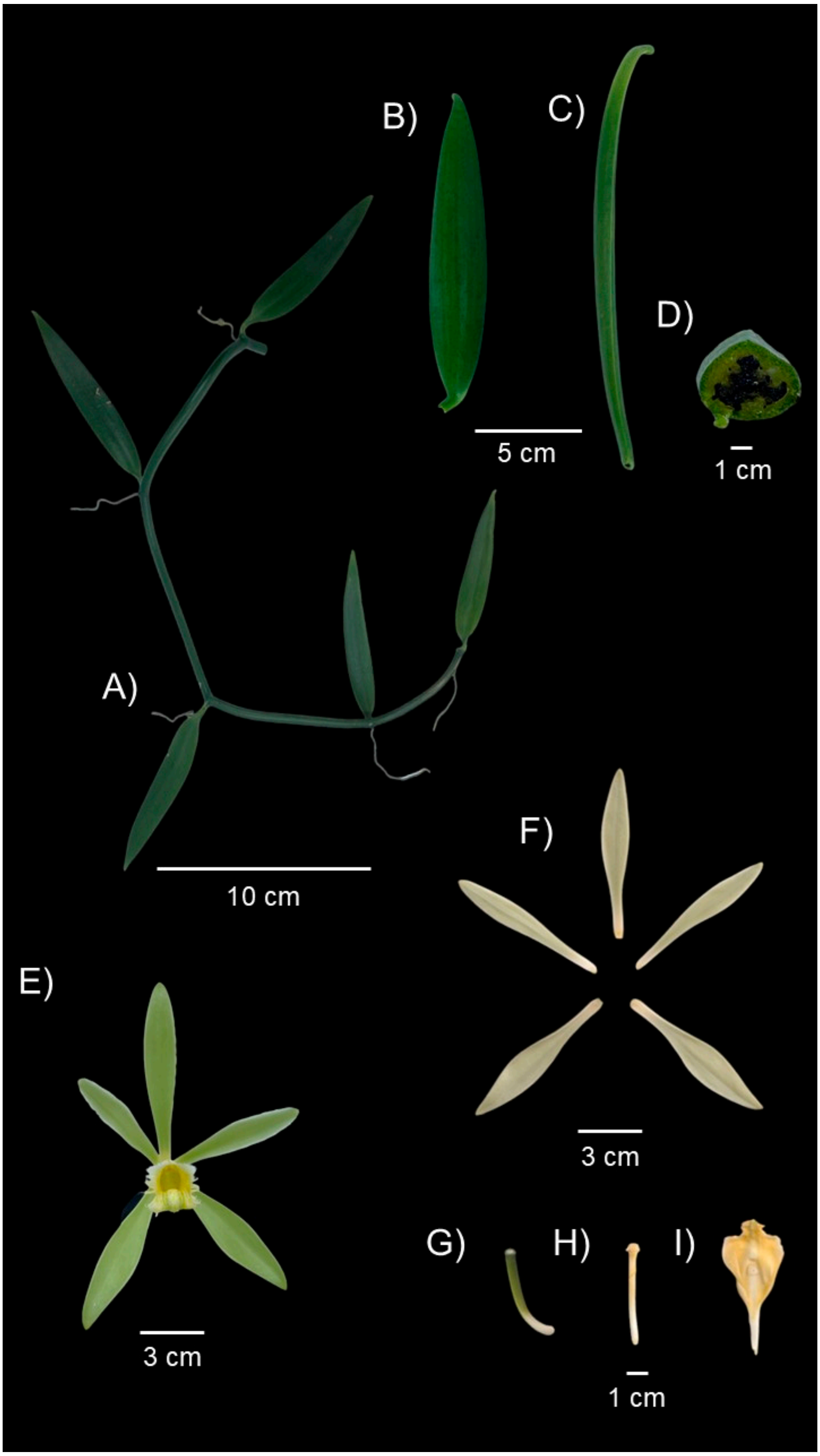

Figure S1. Morphological datasheet of accession ITTUX0033. A) Stem, B) Leaf, C) Fruit, D) Fruit in cross section, E) Flower, F) Sepals and petals, G) Ovary, H) Column and I) Labellum.

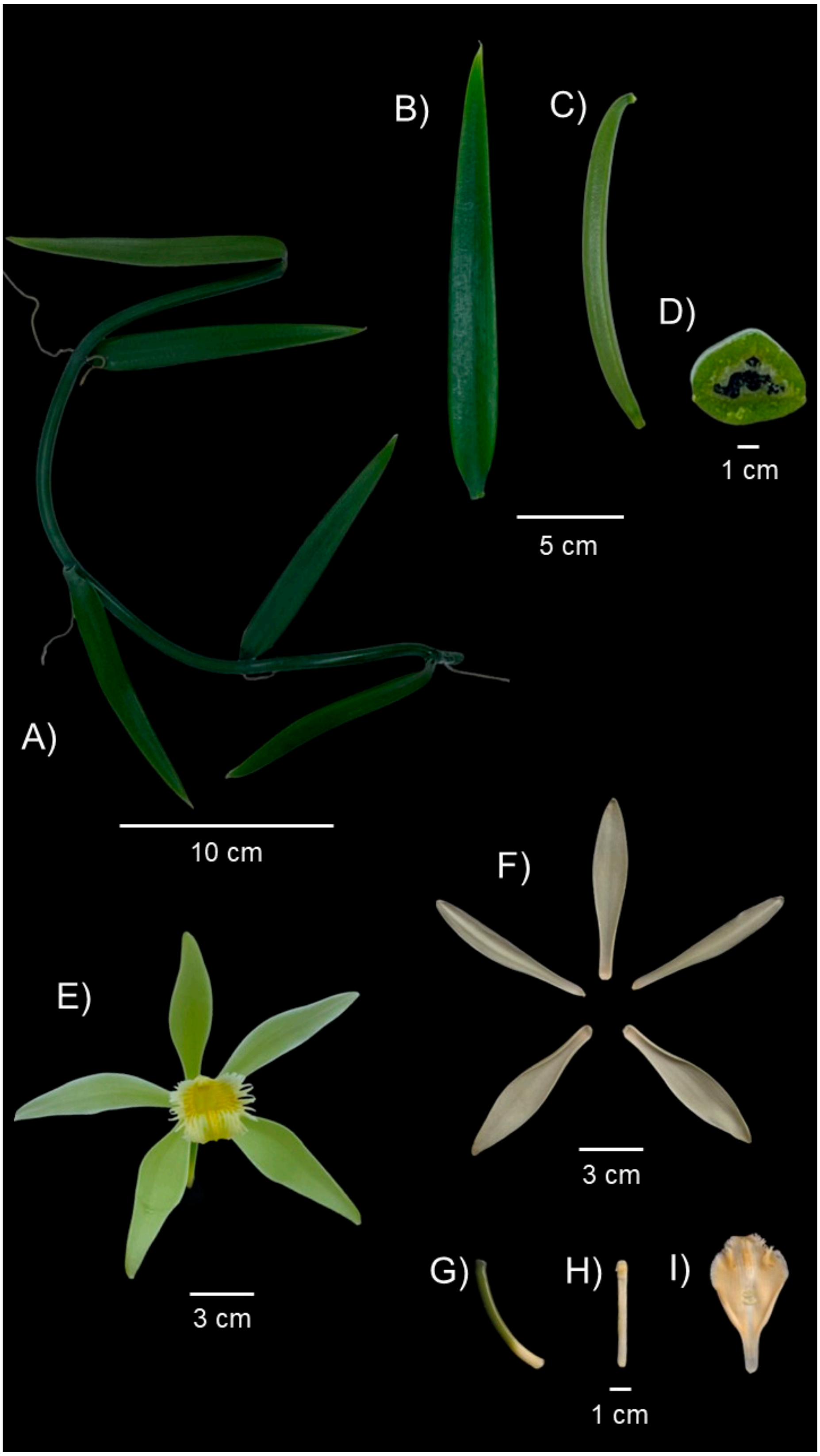

Figure S2. Morphological datasheet of accession ITTUX0037. A) Stem, B) Leaf, C) Fruit, D) Fruit in cross section, E) Flower, F) Sepals and petals, G) Ovary, H) Column and I) Labellum.

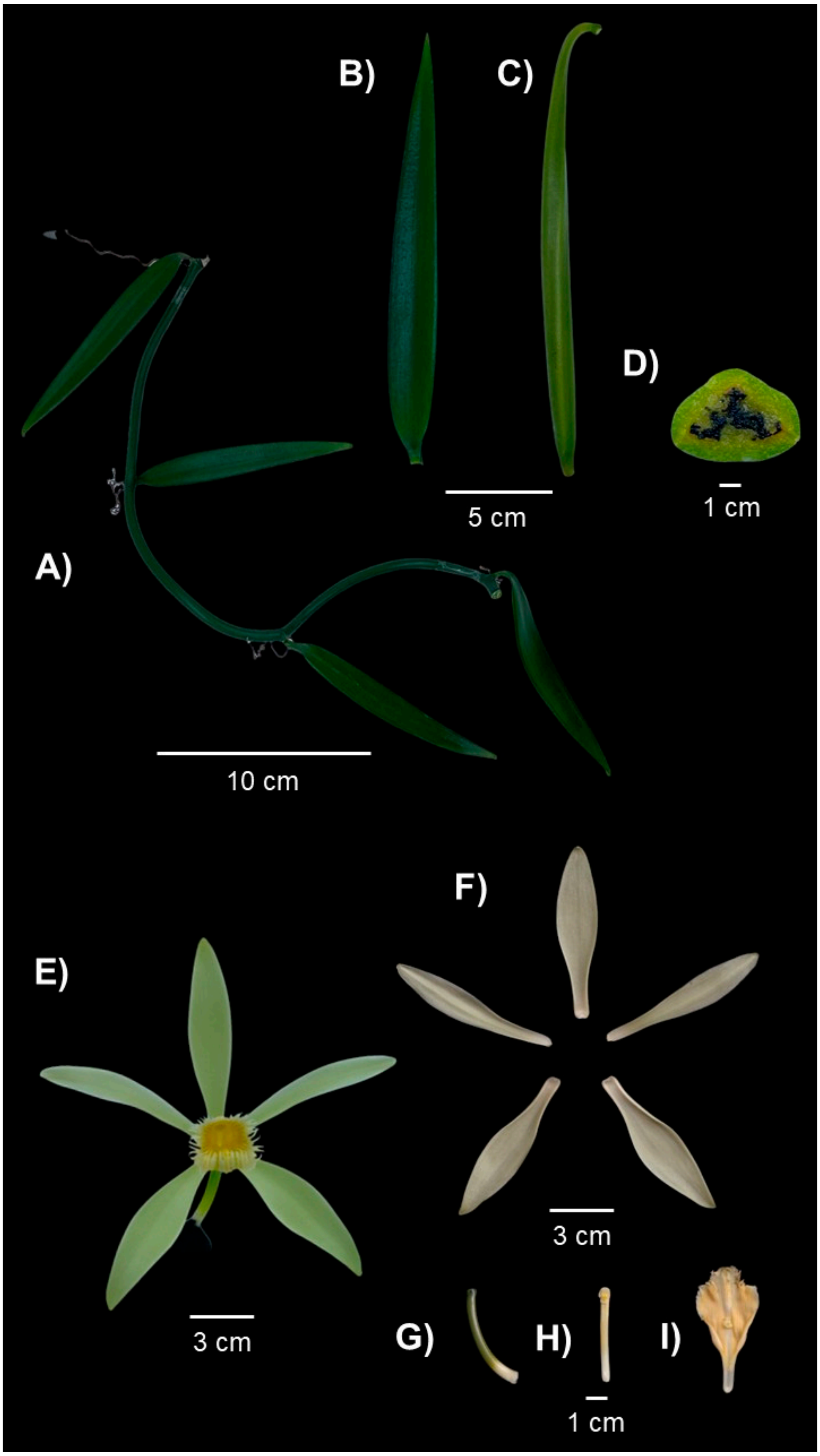

Figure S3. Morphological datasheet of accession ITTUX0066. A) Stem, B) Leaf, C) Fruit, D) Fruit in cross section, E) Flower, F) Sepals and petals, G) Ovary, H) Column and I) Labellum.

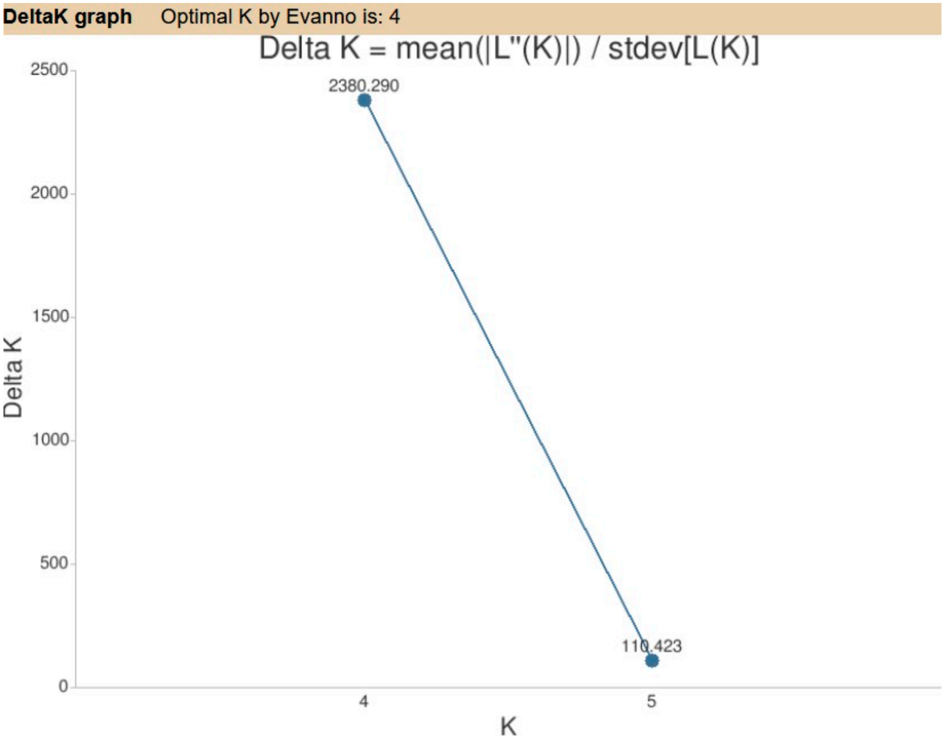

Figure S4. DeltaK graph shows the best K as determined by STRUCTURE HARVESTER.
